# A Novel *SPG7* Gene Pathogenic Variant in a Cypriot Family With Autosomal Recessive Spastic Ataxia

**DOI:** 10.3389/fgene.2021.812640

**Published:** 2022-01-13

**Authors:** Christina Votsi, Antonis Ververis, Paschalis Nicolaou, Yiolanda-Panayiota Christou, Kyproula Christodoulou, Eleni Zamba-Papanicolaou

**Affiliations:** ^1^ Neurogenetics Department, The Cyprus Institute of Neurology and Genetics, Nicosia, Cyprus; ^2^ Neurobiology Department, The Cyprus Institute of Neurology and Genetics, Nicosia, Cyprus; ^3^ Neuroepidemiology Department, The Cyprus Institute of Neurology and Genetics, Nicosia, Cyprus

**Keywords:** Cypriot family, novel missense variant, paraplegin, spastic ataxia, *SPG7* gene

## Abstract

The *SPG7* gene encodes the paraplegin protein, an inner mitochondrial membrane—localized protease. It was initially linked to pure and complicated hereditary spastic paraplegia with cerebellar atrophy, and now represents a frequent cause of undiagnosed cerebellar ataxia and spastic ataxia. We hereby report the molecular characterization and the clinical features of a large Cypriot family with five affected individuals presenting with spastic ataxia in an autosomal recessive transmission mode, due to a novel *SPG7* homozygous missense variant. Detailed clinical histories of the patients were obtained, followed by neurological and neurophysiological examinations. Whole exome sequencing (WES) of the proband, *in silico* gene panel analysis, variant filtering and family segregation analysis of the candidate variants with Sanger sequencing were performed. RNA and protein expression as well as *in vitro* protein localization studies and mitochondria morphology evaluation were carried out towards functional characterization of the identified variant. The patients presented with typical spastic ataxia features while some intrafamilial phenotypic variation was noted. WES analysis revealed a novel homozygous missense variant in the *SPG7* gene (c.1763C > T, p. Thr588Met), characterized as pathogenic by more than 20 *in silico* prediction tools. Functional studies showed that the variant does not affect neither the RNA or protein expression, nor the protein localization. However, aberrant mitochondrial morphology has been observed thus indicating mitochondrial dysfunction and further demonstrating the pathogenicity of the identified variant. Our study is the first report of an *SPG7* pathogenic variant in the Cypriot population and broadens the spectrum of *SPG7* pathogenic variants.

## Introduction

Hereditary cerebellar ataxias (HCA) and hereditary spastic paraplegias (HSP) comprise two groups of genetically and clinically heterogeneous disorders with more than 70 genes and loci reported for each group (Parodi et al., 2018). Pure and complex clinical forms for both groups as well as a considerable overlap have been described (Tesson et al., 2015; Bereznyakova and Dupre, 2018). Recent advancements in genetic diagnosis such as the use of next generation sequencing (NGS), enabled the association of new genes with these disorders and added to the phenotypic expansion of known genes. Therefore, the existing overlap has been strengthened and the new concept of spastic ataxia (SA) spectrum has been defined (Parodi et al., 2018).

Several genes initially classified as HSP or HCA have now been considered as SA genes. The *SPG7* gene with more than 100 reported pathogenic variants, was initially linked to pure and complicated HSP with cerebellar atrophy, and now represents a frequent cause of undiagnosed cerebellar ataxia and SA (Lallemant-Dudek and Durr, 2020). It encodes the paraplegin protein, a member of the ATPases AAA family that is located in the inner mithochondrial membrane and is implicated in other mitochondrial proteins processing (Wedding et al., 2014). Functional studies aiming at understanding the pathogenetic mechanisms of identified variants have linked the protein to mtDNA homeostasis (Wedding et al., 2014).

We hereby describe the genetic investigation of a large Cypriot family with five affected individuals presenting with SA. Whole exome sequencing enabled the identification of a novel homozygous missense variant in the *SPG7* gene. We also provide detailed clinical descriptions of all affected individuals and highlight some intrafamilial phenotypic variation.

## Materials and Methods

### Samples

**FIGURE 1:**
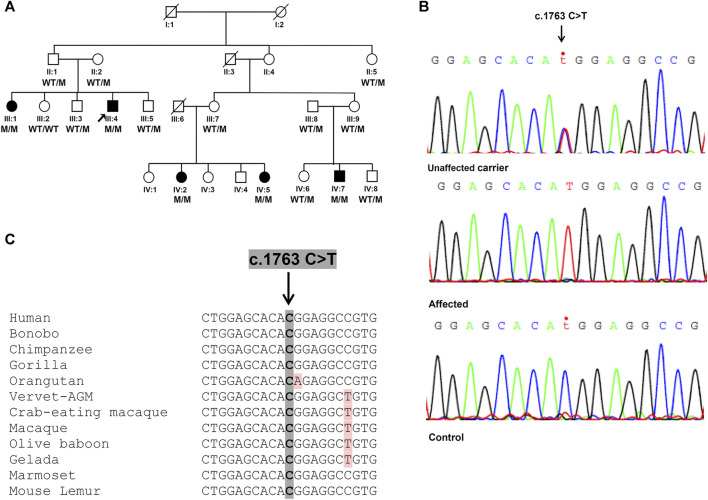
Identification of the *SPG7* novel variant. **(A)** Family pedigree with segregation analysis (WT, wild-type, M, mutant). The arrow indicates the proband. **(B)** Sanger sequencing electropherograms. Unaffected carrier = II:1, affected = III:4 and control = non-related healthy individual. **(C)** Conservation of identified variant. The DNA sequence of the *SPG7* gene region encompassing the identified variant (c.1763C > T; p.Thr588Met) is highly conserved in various mammals.

A four-generation family with 24 members and five patients (two males, three females) in two generations (Figure 1A) was included in the study. The grandparents originate from the same village; however, no relationship has been reported. Detailed clinical histories were obtained from all patients and they were subjected to neurological and neurophysiological evaluation by the CING neurologists. Brain MRI has been performed only at the early stages of the disease for three patients (III:4, IV:5, and IV:7). Blood samples were obtained from individuals II:1, II:2, II:5, III:1, III:2, III:3, III:4, III:5, III:7, III:8, III:9, IV:1, IV:2, IV:5, IV:6, IV:7, and IV:8. DNA was extracted from all available samples using standard salting out procedures.

### Ethics Statement

This study has been approved by the Cyprus National Bioethics Committee (ΕΕΒΚ/ΕΠ/2013/09 and ΕΕΒΚ/ΕΠ/2013/28). Written informed consent was obtained from all study participants. Written informed consent was obtained from the individuals for the publication of any potentially identifiable images or data included in this article.

### Molecular Analyses

#### Whole Exome Sequencing and Variant Filtering

WES was carried out on the proband as described previously (Votsi et al., 2014), followed by variant filtering: a preliminary filtering excluding variants with a minor allele frequency < 0.05 in 1,000 genomes, ExAC (Exome Aggregation Consortium) and EVS (Exome Variant Server) control databases was initially performed. Further *in silico* analysis focused on a gene panel encompassing HCA and HSP genes. Filtering criteria such as the expected genotype (homozygous/heterozygous), the coverage (>20x), the percentage of the alternative allele (>30%), the severity and the effect prediction according to Sift, Condel and Polyphen tools, were applied in order to prioritize the most significant variants to be further investigated for family segregation, with Sanger sequencing. Pathogenicity prediction was performed for a single novel pathogenic variant cosegregating with the disease in the family. Twenty one additional *in silico* tools or algorithms (including the widely used CADD, PROVEAN, Mutation Taster, DANN and other) provided by the VarCards database (Li et al., 2018) were used.

#### Sanger Sequencing

Primers amplifying the sequence encompassing the selected novel candidate variant, were designed by us. Amplification products were sequenced in both directions using the Big Dye Terminator v1.1 Cycle Sequencing kit [Applied Biosystems (ABI), California, United States]. Sequence traces were automatically compared with the normal region sequences as listed in the GenBank database using the Seqscape software (ABI).

#### Cell Lines

Lymphoblastoid cell lines (LCLs) were previously established using the standard Epstein Barr virus (EBV) infection procedure, for a couple of patients and independent control individuals after informed consent. They were cultured in RPMI 1640 medium (Biosera, Nuaille, France), supplemented with 10% FBS (ThermoFisher, Massachusetts, United States), and 50 U/ml of Penicillin/Streptomycin (Biosera).

Human SH-SY5Y were cultured in Dulbecco’s Modified Eagle’s medium (Biosera) supplemented with 10% FBS, 2 mM Glutamine (ThermoFisher), 50 U/ml Penicillin and 50 mg/ml Streptomycin.

All cell lines were kept at 37°C in 5% CO_2_.

#### RNA Analysis

Total RNA was extracted from patients and independent control individuals LCLs, using the RNeasy Mini Kit (Qiagen, Hilden, Germany). cDNA was synthesized using the Protoscript First Strand cDNA Synthesis Kit (New England Biolabs, Ipswich, MA, United States). The synthesized strands were used as substrates for RNA expression studies and cloning of the *SPG7* gene. Three independent real-time PCR experiments of three technical replicates for each sample, using TaqMan probes for the *SPG7* (Hs00275795_m1) and two housekeeping genes [*GAPDH* (Hs99999905_m1) and *B2M* (Hs00187842_m1)] were performed using the QuantStudio 7 Flex instrument (ABI). The QuantStudio Real-Time PCR software was used for data analysis and the Student’s *t*-test was used for the statistical analysis of the independent experiments derived values. A *p*-value < 0.05 was set as a threshold for statistical significance.

#### Plasmid Construction and Cell Transfection

The cDNA samples of a control individual and the proband were used as templates in order to amplify the wild-type (WT) and mutant *SPG7* ORFs, respectively. Appropriate primers sets containing specific restriction sites in order to insert the amplified fragments into the p-EGFP-N1 vector were designed by us and are available upon request. Sanger sequencing was employed in order to confirm that all plasmid constructs have been free of any new nucleotide changes in the *SPG7* cDNA. SH-SY5Y cell lines were then transfected with these constructs with the use of Lipofectamine 3000 (ThermoFisher) following the manufacturer’s instructions. Cells were harvested 48 h after transfection for protein extraction.

#### Protein Analysis

In order to perform expression studies, protein was extracted from the patients’ and controls’ LCLs as well as the transfected SH-SY5Y cells as described previously (Ververis et al., 2020). After running in an SDS-PAGE polyacrylamide gel, proteins were transferred to Hybond-P hydrophobic polyvinylidene difluoride (PVDF) membranes (Millipore, Germany). Membranes were blocked for 3 h (LCLs) or 1 h (SH-SY5Y) in 5% non-fat dry milk in phosphate buffered saline (PBS)/Tween, at room temperature, followed by overnight incubation at 4°C with the primary antibodies for the protein of interest and a housekeeping control [rabbit anti-SPG7 (Sigma Aldrich Taufkirchen, Germany)/1:6,500 for LCLs, rabbit anti-GFP (Proteintech, Illinois, United States)/1:5,000 for SH-SY5Y, mouse anti-β-ACTIN (Sigma-Aldrich)/1:4,000 for LCLs, mouse anti-vinculin (7F9) (Santa Cruz Biotechnology, Heidelberg, Germany)/1:1,000 for SH-SY5Y]. The next day membranes were washed 3 × 10 min in PBS/Tween and incubated with the appropriate secondary antibodies for 1 hour. Three washes followed and proteins signals were visualised using the LumiSensor Chemiluminescent HRP Substrate Kit (Genscript, New Jersey, United States), in a UVP BioSpectrum Imaging System (UVP, California, United States). Three independent experiments were carried out for each case. The ImageJ 1.51j8 software was used for data analysis.

#### Immunofluorescence

SH-SY5Y cells were grown on coverslips. After 24 h they were transiently transfected with the pEGFP-N1 *SPG7* ^WT^ and mutant constructs with the use of Lipofectamine 3000 (ThermoFisher) following the manufacturer’s instructions. After 48 h, cells were fixed for 10 min at RT with 4% w/v paraformaldehyde in PBS, permeabilised with Methanol for 10 min at −20°C and quenched for 10 min at RT with 50 mM NH_4_Cl in PBS. Cell mitochondria were stained using the AIF antibody (Cell Signaling Technology, Leiden, Netherlands) and nuclei using Hoechst 33342 (ThermoFisher). Coverslips were mounted with Dako Fluorescent Mounting Medium (Agilent, California, United States). Images were captured with a Zeiss fluorescent microscope using the Axiovision software. Colocalization index was quantified by Manders’ coefficient using Coloc2 plugin for ImageJ, and two-tailed *t* test was used to assess the *p* value. Mitochondrial morphology parameters were assessed with the use of a macro titled “mitochondrial morphology,” developed by the Chu Lab for ImageJ as previously described (Dagda et al., 2009). One-way ANOVA analysis was conducted for the statistical evaluation of the mitochondrial morphology parameters.

## Results

### Clinical Picture

The proband (III:4) developed balance difficulties during secondary school (16 years old) and by the age of 31 he presented spastic paraparesis. His elder sister (III:1) had a disease onset much later at 46, whereas the younger son of their cousin (IV:7) had an early onset, at 17. The two affected cousins (IV:2 and IV:5) had an onset between 23–25. All patients initially presented with gait unsteadiness and impaired balance which gradually deteriorated. They developed spasticity in the lower limbs, ataxic spastic gait and lower limb proximal weakness (Table 1). Dysarthria, bilateral nystagmus, dysmetria and dysdiadochokinesia, as well as bilateral extensor plantar reflex and clonus were observed in all patients. Progressive external ophthalmoplegia (PEO), pes cavus foot deformity, urinary incontinence and upper limbs weakness at advanced stage with facial weakness, double vision and mild neuropathy were also observed in some of the patients (Table 1). Patient III:1 also suffers from abdominal pains and gastrointestinal hemorrhages for many years, but most probably these derive from a non-neurological disease. Electrophysiological evaluation of the patients with nerve conduction studies revealed sensory-motor axon neuropathy in two patients (III:1, IV:5). Somatosensory evoked potentials examinations were within normal limits in the majority of the patients. Bilateral optic pathway dysfunction in patients III:4 and IV:7 has been indicated at advanced stages of the disease through visual evoked potentials examination. Brain MRI at the early stages of the disease (performed on three patients) revealed cerebellar atrophy. Muscle biopsy performed on the proband at the early stages of the disease, revealed a normal mitochondria number and structure. Overall, the progression of the disease was slow and the patients became wheel-chair bound on average 20 years after the disease onset. The first clinical impression had been recessive cerebellar ataxia with spasticity and more recently it has been redefined to SA.

**TABLE 1:** Clinical features of the patients.

Patient	III:1	III:4	IV:2	IV:5	IV:7
Gender	Female	Male	Female	Female	Male
Age at onset	46	16	23	25	17
First symptom(s)	Unst/GD	Unst/GD	Unst/GD	Unst/GD	Unst/GD
Age at examination	48	32	45	39	18
Mean follow up duration	30	38	11	12	32
Gait	Ataxic-spastic	Ataxic-spastic	Ataxic-spastic	Ataxic-spastic	Ataxic-spastic
Muscle weakness	LL, facial	LL, facial	LL, UL, facial	LL, UL	LL, Facial
Muscle wasting	−	−	−	−	−
Muscle tone UL	Increased	Normal	Normal	Normal	Normal
Muscle tone LL	Increased	Increased	Increased	Increased	Increased
Tendon reflexes	Brisk	Brisk	Brisk	Brisk	Brisk
Extensor plantar reflex	Bilateral	Bilateral	Bilateral	Bilateral	Bilateral
Cerebellar signs	Dysmetria and DDK	Dysmetria and DDK	Dysmetria and DDK	Dysmetria and DDK	Dysmetria and DDK
Dysarthria	+++	++	++	++	++
Dysphagia	++	−	−	−	−
Nystagmus	+	+	+	+	+
Sensory deficit	−	+^a^	−	−	−
Skeletal abnormalities	−	Pes cavus	−	−	Pes cavus
Ophthalmological signs	PEO	PEO and diplopia	Diplopia	−	PEO and diplopia
Optic Atrophy	−	+	−	−	−
Extrapyramidal symptoms	−	−	−	−	−
Peripheral Neuropathy	+	−	−	+	−
Dementia/Psychosis	−	−	−	−	−
Cognitive impairment	−	−	−	−	−
Bladder disturbance	UI	Mild UI	−	UI	−
Hearing loss	−	−	−	−	−
Respiratory difficulties	+++	−	−	−	−
Brain MRI findings	Not available	CA	Not available	CA	CA

a Slightly reduced vibration and proprioception; CA, cerebellar atrophy; DDK, dysdiadochokinesia; LL, lower limbs; PEO, progressive external ophthalmoplegia; Unst/GD, Unsteadiness/Gait Difficulties; (−), absent; (+), mild (or active); (++), moderate; (+++), severe; UL, upper limbs; UI, urinary incontinence

### Genetic Analysis

A total of 71,696,896 reads, an average coverage of >20x for 92% of the target regions and 43.500 variants were identified through WES of the proband. After initial exclusion of variants with a minor allele frequency >0.05 in public databases, further analysis considering the HSP/HCA genes panel, enabled a significant reduction of the candidate variants to nine (Supplementary Table S1). Further evaluation considering the genotype, the variant consequence and severity, resulted in the identification of the single homozygous missense variant c.1763C > T, p.Thr588Met in the *SPG7* gene (Figure 1B), which has been also confirmed with family segregation analysis. Nucleotide and protein position of the reported variant are based on RefSeq accession numbers NM_003119.2 and NP_003110.1. This is a novel variant neither registered in gnomAD (gnomad.broadinstitute.org/), ExAC, 1,000 genomes, the ClinVar nor the LOVD databases. It has been also excluded from more than 200 control Cypriot chromosomes using Sanger sequencing. This variant results in the replacement of a neutral by a hydrophobic amino acid, and is located in the conserved peptidase M41 domain (Figure 1C) where many other missense pathogenic variants have been described. It is predicted to affect the protein function causing deleterious consequences. High pathogenicity scores thus enabling the characterizations “deleterious” or “damaging” which denote the most severe possible effects, resulted from 20 prediction algorithms or tools which are based either on single or diverse annotation methods. These included the Sift (0.001), the Provean (−4.94), the CADD (34), the DANN (0.999), the MutationTaster (1.0), the FATHMM (−1.72), the LRT (0.0), and other scores.

### RNA and Protein Expression Studies

**FIGURE 2:**
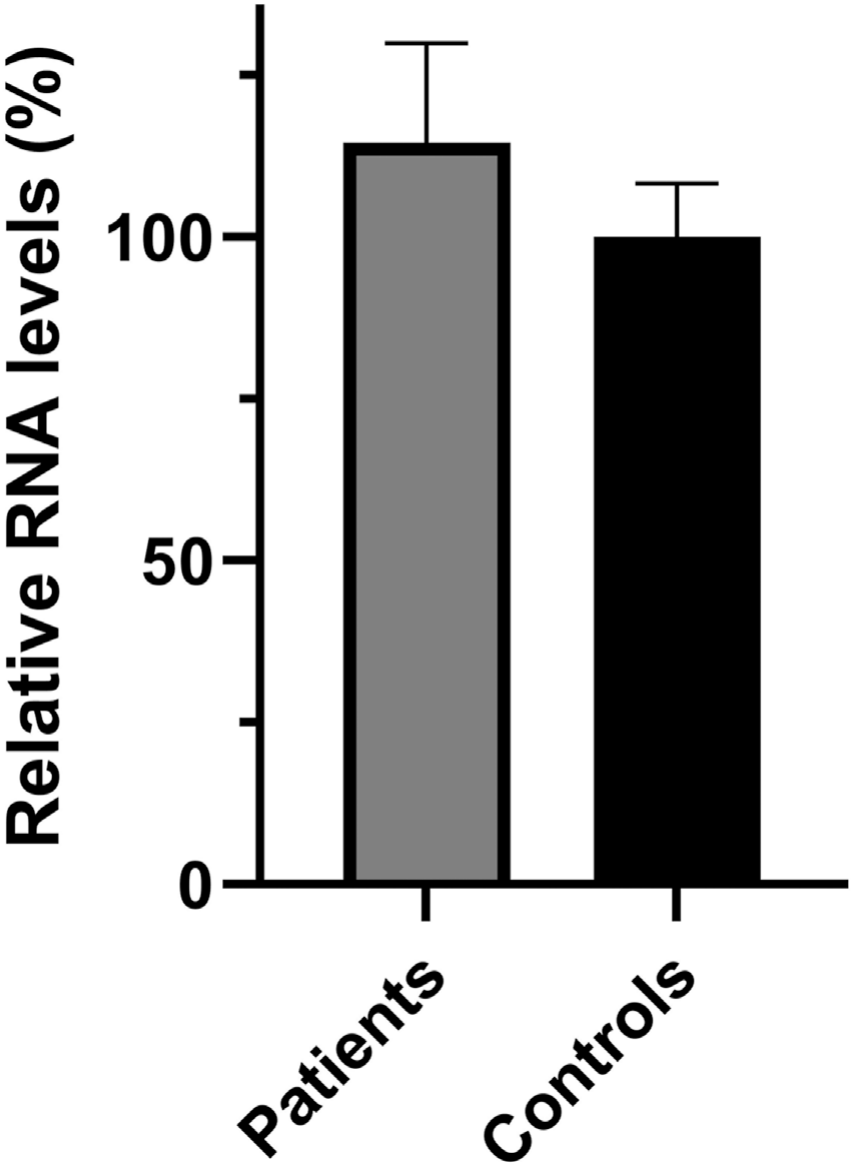
Relative *SPG7* mRNA expression levels in lymphoblastoid cell lines derived from patients and control individuals. qPCR revealed that the *SPG7* gene expression has no significant difference between the patients (average 114%) and the controls (set to 100%), *p* > 0.05. Values were obtained after normalization with the *GAPDH* and *B2M* housekeeping genes. Data are represented as the mean of three independent triplicate experiments ± SD.

**FIGURE 3:**
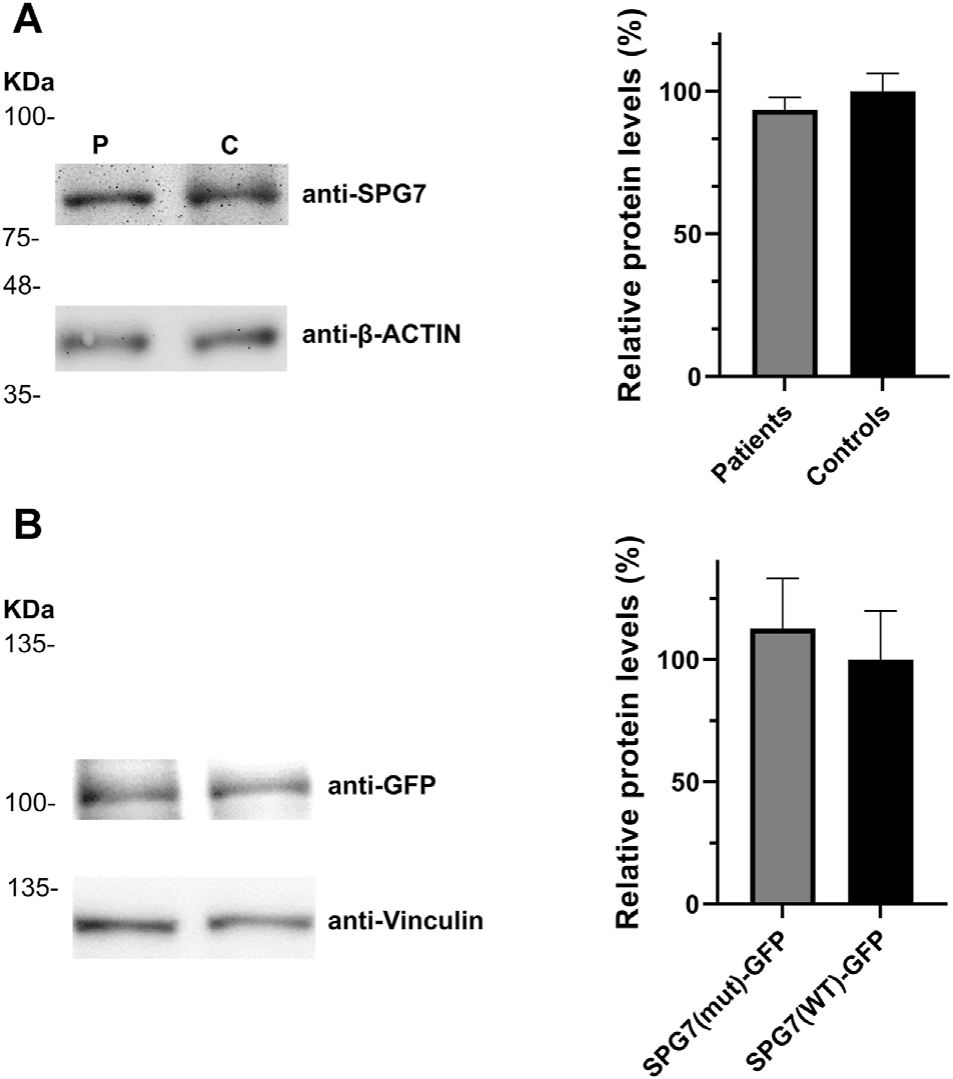
Relative SPG7 protein expression levels in cells expressing the wild-type and the pathogenic variant. (**A)** Western blot using an anti-SPG7 antibody revealed similar protein expression between the group of patients with spastic ataxia (average 94%) and the group of control individuals (set to 100%) lymphoblastoid cell lines. Values were obtained after normalization with the β-actin housekeeping protein. Data are represented as the mean of three independent experiments ± SE. **(B)** Western blot using an anti-GFP antibody revealed similar SPG7-GFP protein expression in SH-SY5Y cells, transfected with the p-EGFP-N1 constructs expressing SPG7^T588M^-GFP (average 113%) or SPG7^WT^-GFP (set to 100%). Values were obtained after normalization with the vinculin housekeeping protein. Data are represented as the mean of three independent experiments ± SE.

In addition to the pathogenicity prediction results for the novel *SPG7* c.1763C > T variant, we investigated a possible effect on the RNA and protein expression. To compare the expression of SPG7^WT^ and SPG7^T588M^, RNA and protein extracts derived from two available patients and three control individuals LCLs were used. qRT-PCR lead to the observation of similar expression levels between the patients and controls (Figure 2) thus excluding any effect of the variant on the RNA expression levels in this tissue. Western blot analysis using an anti-SPG7 antibody also did not indicate a significant difference between patient and control (Figure 3A). To further investigate if this was a tissue specific result we also performed protein expression studies using an *in vitro* model, the human derived neuroblastoma SH-SY5Y cell lines that had been transfected with the p-EGFP-N1 constructs expressing SPG7^WT^ and SPG7^T588M^. Similar to the LCLs, western blot analysis using an anti-GFP antibody (thus detecting only the exogenous SPG7), did not indicate any significant difference between the expression of the mutant and the WT SPG7 in these cells (Figure 3B).

### Protein Localization and Mitochondrial Morphology

**FIGURE 4:**
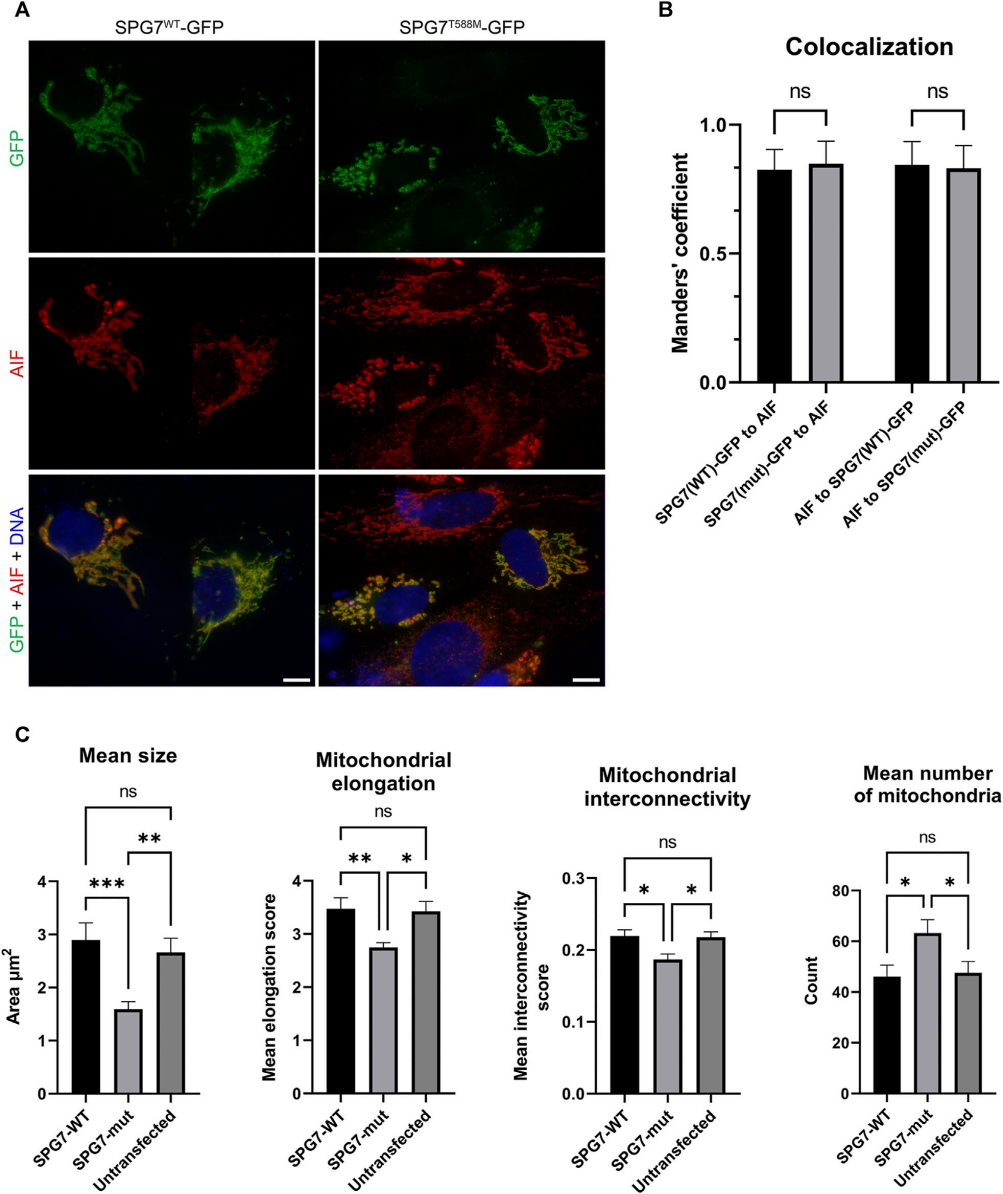
Intracellular localization of SPG7^WT^-GFP and SPG7^T588M^-GFP and mitochondrial morphology assessment. **(A)** SPG7^T588M^-GFP retains colocalization with mitochondria, in similar fashion with SPG7^WT^-GFP. Green color represents the SPG7-GFP, red color represents mitochondria and blue color represents the nuclei. Scale bars are 10 μm. **(B)** Graph displaying Manders’ coefficient quantification (fraction SPG7^WT^-GFP or SPG7^T588M^-GFP colocalizing with AIF, and vice versa) calculated from at least 20 fluorescence microscopy images for each isoform. Error bars are given by the SD and *n* > 30 for each condition. **(C)** Graphs displaying morphological characteristics of mitochondria: size, elongation, interconnectivity and number, in WT (SPG7^WT^-GFP) and mutant (SPG7^T588M^-GFP) transfected SH-SY5Y cells as well as in untransfected cells, calculated from at least 20 fluorescence microscopy images for each condition. Error bars are given by the SEM and *n* > 30 cells for each condition (* refers to *p* < 0.05, ** to *p* < 0.01, and *** to *p* < 0.001 statistical significance, while ns corresponds to non-significant for the comparisons shown).

We also performed immunofluorescence experiments using the transfected SH-SY5Y cells, in order to investigate the localization of the SPG7^WT^ and SPG7^T588M^ proteins and assess the mitochondrial morphology of mutant and WT cells by evaluating four well documented parameters (Dagda et al., 2009; Wiemerslage and Lee, 2016). The resulting data showed that the mutant protein retains its localization to mitochondria similar to the WT (Figures 4A,B), thus indicating that possibly the novel variant does not affect protein localization. Regarding mitochondrial morphology, it has been observed that mitochondria in mutant cells were smaller and less elongated with a low degree of interconnectivity (Figures 4A,C), which are typical signs of a dysfunctional mitochondrial network. In contrast, mitochondria in WT cells were longer, elongated and interconnected (*p* < 0.05; Figures 4A,C), indicating a healthy mitochondrial network. Furthermore, the mitochondria mean number of mutant cells (Figure 4C) was significantly higher compared to the WT, and that accompanied by a smaller mitochondrial size indicates mitochondrial fragmentation.

## Discussion

We hereby describe the clinical picture and the genetic investigation of a large Cypriot family with SA. Before the advent of WES, the family had been extensively studied and excluded in the past (more than 15 years ago) for linkage to HCA genes and loci (guided from the first clinical impression and that period relevant literature), as well as pathogenic variants previously reported in the Cypriot population (Nicolaou et al., 2008; Zamba-Papanicolaou et al., 2009; Votsi et al., 2014).

It is widely accepted that the advent of NGS strategies, including the targeted sequencing panels, the WES and the whole-genome sequencing (WGS), improved dramatically the genetic diagnosis of clinically and genetically heterogeneous diseases such as the HCAs and HSP. NGS has also enabled the identification of many novel genes and variants (Coutelier et al., 2018; Elert-Dobkowska et al., 2019; Krygier and Mazurkiewicz-Bełdzińska, 2021; Sahin and Saat, 2021). Through a WES approach followed by *in silico* targeted gene analysis, the present study describes the first identification of a pathogenic variant in the *SPG7* gene in the Cypriot population. The successful efficacy of this approach in the study of HCAs and HSPs has been well documented (Coutelier et al., 2018; Sahin and Saat, 2021; Saputra and Kumar, 2021). Compared to other diagnostic approaches such as the classic Sanger sequencing based on phenotype-oriented gene prioritization, or even the NGS gene targeted panel sequencing, the exome targeted capture has several advantages. Gene prioritization based on phenotypic presumptions is abolished, and there is no need to redesign a panel in case new HCA or HSP genes are published. Moreover, in the context of the HCA and HSP heterogeneity, a broader range of genes must be allowed to be tested, including genes linked with other types of transmission or other neurological diseases which have overlapping features with HCA or HSP. This approach captures variants in genes associated with various transmission modes or variants that would not be identified by a typical HCA or HSP panel. However, it might be more expensive than a targeted panel analysis with a relatively lower coverage provided. The major limitation of both the targeted panel and WES compared to the WGS approach, which provides longer reads, has been their inability to detect repeat expansions, deep intronic variants or CNVs (Coutelier et al., 2018; Krygier and Mazurkiewicz-Bełdzińska, 2021; Saputra and Kumar, 2021). Therefore, for undiagnosed patients excluded from small scale variants through gene panel analysis or WES, the use of WGS could be a promising option. Thus far, WGS has contributed to discovering ataxia (Cortese et al., 2019) and other disease novel tandem repeat expansions (Bahlo et al., 2018; Kim et al., 2019) and is expected to identify additional new ataxia-causative expansions in the future. Further advances in NGS techniques and bioinformatics analysis are promising towards better detecting pathogenic variants and expansions even from the analysis of short-read data.

Pathogenic variants in the *SPG7* gene had been initially linked to pure and complicated HSP, whereas lately they have been considered as frequent causes of undiagnosed cerebellar ataxia and SA cases. In the United Kingdom, *SPG7* variants have been reported as the fourth more common cause of the genetic ataxias in general and the second of the recessive ataxias (Hewamadduma et al., 2018). A variety of variants including several missense, frameshift, nonsense, small insertions, deletions, duplications, macro deletions and splice site variants have been described. More recently, a deep intronic heterozygous cryptic splice variant has been reported to cause SA in combination with a heterozygous missense variant, thus suggesting the re-examination of existing cases characterized as dominant *SPG7* variants (Verdura et al., 2020).

The core clinical presentation of the Cypriot family patients is similar to other described cases. The majority of their features correspond to >50% of the worldwide described cases (Hewamadduma et al., 2018). However, within the family some phenotypic differences were observed, with the age of onset being the most significant. It has been reported that the localization of a mutation in relation to the different functional domains of the protein affects the age of onset. More specifically, homozygous mutations in the M41 peptidase domain have been correlated to an earlier disease onset compared to mutations in a non-functionally assigned domain (Hewamadduma et al., 2018). This novel missense *SPG7* c.1763C > T variant is located within the M41domain, and is associated with a variable age of disease onset (ranging from 16–46 years). Patient III:1 had a very later age of onset compared to the other patients, and the two males had an earlier onset compared to the other females. Moreover, only patient III:1 presents with respiratory difficulties and dysphagia, as well as a more severe cerebellar dysarthria. Additional symptoms which are not common for all the patients such as optic atrophy, sensory deficit, pes cavus deformity, facial weakness, double vision, peripheral neuropathy and urinary incontinence have been recorded. Moreover, PEO is present in 3/5 patients, and it has been referred as one of the rare findings in the majority of described *SPG7* cohorts (Hewamadduma et al., 2018; Verdura et al., 2020). To our knowledge, similar phenotypic differences in patients sharing an identical *SPG7* pathogenic variant, have been observed, mostly between different families and more rarely between members of the same family (Van Gassen et al., 2012; Hewamadduma et al., 2018). Such differential phenotypic expression could be attributed to the contribution of other genetic, epigenetic or environmental factors.

The identified novel *SPG7* variant has been predicted to cause deleterious consequences with almost all the prediction tools. There are many possible effects of a missense variant including protein folding, stability and flexibility alterations, binding processes prevention, subcellular localization and expression alterations. One or a combination of such effects might cause an abnormal protein function (Zhang et al., 2012). To investigate the possible expression and subcellular localization alterations, we employed the patient/control derived LCLs (only for expression) and the *in vitro* model neuroblastoma SH-SY5Y cell lines. The SH-SY5Y cell lines is a well-established model that has been used for the investigation of various neurodegenerative disorders, including ataxia and spastic paraplegia types (Bolinches-Amoros et al., 2014; Wagner et al., 2019; Morani et al., 2020). However, to our knowledge, this is the first time that they are employed for the study of an *SPG7* relevant disease. Furthermore, there are no other reports for the investigation of the effect of missense *SPG7* variants in LCLs. There are limited reports for differential expression in other cells, in most of which a missense variant was in compound heterozygosity with another type of variant and therefore its contribution (if any) was not clear. Increased RNA and protein expression was observed in muscle cells of a group of patients carrying *SPG7* compound heterozygous variants (nonsense and missense) (Pfeffer et al., 2014). Increased protein expression was also identified in olfactory neurosphere-derived cells, isolated from patients carrying the common missense p.Ala510Val variant in compound heterozygosity with other variants (Wali et al., 2020), whereas decreased protein expression was identified in fibroblasts of a patient heterozygous for a missense variant and a deep intronic cryptic splice variant (Verdura et al., 2020). Another study reported paraplegin accumulation in post-mortem cerebellar and cerebral cortex neurites of a patient homozygous for the common p.Ala510Val variant; however, it was not clear whether this observation was due to the protein overexpression or mislocalization (Thal et al., 2015). Our experimental data indicate that the reported variant does not affect the expression of either the RNA or the protein in both tested cell lines. Localization of the mutant protein remains unaffected as well.

Aberrant mitochondrial morphology representing an unhealthy mitochondrial network has been detected in cells expressing SPG7^T588M^, thus indicating mitochondrial dysfunction and further supporting the predicted pathogenicity of the reported variant. Similar mitochondrial morphology, accompanied by additional types of mitochondrial dysfunction that have been associated with neurodegeneration (including decreased mitochondrial membrane potential, reduced mitochondrial mass, reduced oxidative phosphorylation, reduced ATP content, diminished cellular proliferation and increased mitochondrial oxidative stress) were identified in the olfactory neurosphere-derived cells mentioned above (Wali et al., 2020). Mitochondria defects associated with neurodegeneration have also been described through studies in other cell and animal models lacking paraplegin (Atorino et al., 2003; Ferreirinha et al., 2004; Pareek et al., 2018). In contrast, increased mitochondrial mass and enhanced networks which are referred as typical for a mitochondrial disorder, have been identified in fibroblasts of patients carrying compound heterozygous variants (with at least one of the two being nonsense in the three patients) (Pfeffer et al., 2014).

Based on our findings, we hypothesize that the currently reported *SPG7* variant consequences on the tested cell lines mitochondrial network, are not relevant with protein expression alterations or mislocalization. Other mechanism(s) such as the protein folding, stability and flexibility changes, and protein interaction alterations or a combination of these, might affect protein’s normal function, thus leading to the observed dysfunctional mitochondrial network and perhaps to other dysfunction(s) that remain unknown.

In conclusion, our study is the first report of an *SPG7* pathogenic variant in the Cypriot population. It is a novel variant that broadens the spectrum of *SPG7* pathogenic variants and evidently causes mitochondrial dysfunction. It also provides detailed clinical features of the patients, thus indicating intrafamilial differences, and enabling comparisons with other reported cases. Our findings also further support the usefulness of WES followed by *in silico* targeted gene analysis as a powerful diagnostic tool in the study of clinically and genetically heterogeneous diseases such as the HCAs and HSPs, as well as the suitability of the SH-SY5Y cells for the functional investigation of *SPG7* variants.

## Data Availability

The datasets for this article are not publicly available due to concerns regarding participant/patient anonymity. Requests to access the datasets should be directed to the corresponding author.
