# Supplementary material for: A Novel SPG7 Gene Pathogenic Variant in a Cypriot Family With Autosomal Recessive Spastic Ataxia
Source: Front Genet. 2022 Jan 13;12:812640. doi: 10.3389/fgene.2021.812640 (PMC8793673; doi:10.3389/fgene.2021.812640)
Supplement: Supplementary file 1 [file Table1.DOCX]

**Supplementary Table S1:** List of whole exome sequencing derived candidate variants remaining after filtering

| Gene | Genotype | Consequence | HGVSc | HGVSp | dbSNP ID |
| --- | --- | --- | --- | --- | --- |
| *VPS13D* | het | synonymous_variant | NM_015378.2:c.2583A>G | NM_015378.2:c.2583A>G(p.=) |  |
| *NPHP3-ACAD11, UBA5* | het | upstream_gene_variant |  |  | rs770749604 |
| *PEX6* | het | downstream_gene_variant |  |  | rs368299692 |
| *SPAST* | het | intron_variant | NM_014946.3:c.415+48delG |  |  |
| *MTPAP* | het | splice_region_variant,intron_variant | NM_018109.3:c.1313-3C>T |  | rs201064853 |
| *MACROD1, FLRT1* | het | intron_variant | NM_014067.3:c.517+34934G>C |  | rs139768227 |
| *C12ORF65* | het | synonymous_variant | NM_152269.4:c.243C>T | NM_152269.4:c.243C>T(p.=) | rs140411575 |
| *C12ORF65* | het | missense_variant | NM_152269.4:c.413A>G | NP_689482.1:p.Lys138Arg | rs147328685 |
| *SPG7* | **hom** | **missense_variant** | **NM_003119.2:c.1763C>T** | **NP_003110.1:p.Thr588Met** |  |
